# Vacuolar (H^+^)-ATPase Genes Are Essential for Cuticle and Wing Development in *Locusta migratoria*

**DOI:** 10.3390/genes16020145

**Published:** 2025-01-24

**Authors:** Xiaojian Liu, Xiaoyu Liang, Xuekai Shi, Jianzhen Zhang

**Affiliations:** 1 Shanxi Key Laboratory of Nucleic Acid Biopesticides, Research Institute of Applied Biology, Shanxi University, Taiyuan 030006, China; 2 College of Biological Sciences and Technology, Taiyuan Normal University, Jinzhong 030619, China

**Keywords:** *L* *. migratoria*, vacuolar H^+^-ATPase, wing development, cuticle development, RNA interference

## Abstract

Background/Objectives: Vacuolar (H^+^)-ATPases (V-ATPases) are crucial in several significant biological processes, including intracellular transport, endocytosis, autophagy and protein degradation. However, their role in the growth and development of insects remains largely unknown. This study aimed to explore the molecular and functional properties of *V-ATPases* in *Locusta migratoria*. Methods: LmV-ATPase genes were identified based on the locust transcriptome database and bioinformatics analysis. Quantitative reverse-transcription polymerase chain reaction was used to assess the relative expression of *LmV-ATPases* in different tissues and developmental stages. RNA interference combined with hematoxylin–eosin staining and transmission electron microscopy was used to explore the functions of *LmV-ATPases*. Results: Ten V-ATPase genes were identified in *L. migratoria* and were named *LmV-ATPase A*, *B*, *C*, *D*, *E*, *F*, *G*, *c*″, *d* and *e*, respectively. These genes were highly expressed in the head, integument, gastric caecum, midgut, hindgut, fat body, trachea and ovary. The transcripts of *LmV-ATPases* were expressed in the developmental stages examined (from the 3rd to 5th instar nymphs). The injection of double-stranded RNA (dsRNA) against each *LmV-ATPase* induced high silencing efficiency in the 3rd instar nymphs. Knockdown of *LmV-ATPases* resulted in lethal phenotypes, with visible defects of the wing and cuticle. We further demonstrated that the deformation was caused by the defects of epidermal cells and fewer new cuticles. Conclusions: These findings suggest that *LmV-ATPases* are required for the wing and cuticle development of *L. migratoria*, which could be potential targets for the control of locusts.

## 1. Introduction

Vacuolar (H^+^)-ATPase (V-ATPase or VHA) was first identified in yeast cells, in which its detailed structure and function were investigated [1]. V-ATPase is a conserved enzyme that is found in all eukaryotic organisms, including fungi, plants, worms, insects and mammals [2]. V-ATPase can transport protons into intracellular compartments, which is crucial for pH homeostasis in lysosomes, secretory vesicles, synaptic vesicles and endosomes [3]. Over the past few decades, numerous studies have demonstrated that V-ATPases are essential for many cellular processes, including intracellular trafficking, protein degradation, signal regulation, endocytosis and neurotransmission [4].

A holoenzyme V-ATPase comprises two functional complexes, the cytoplasmic V1 and the membrane-embedded Vo. The V1 domain is composed of subunits A–H and is responsible for ATP hydrolysis. The Vo domain consists of subunits a, d, e, c and c″ (c′ in yeast or Ac45 in higher eukaryotes) and carries out proton transport [5,6]. Some of the V-ATPase subunits exist in several isoforms, which are often expressed in a cell- or tissue-specific manner to fulfill the diverse functions of V-ATPases [6]. In *Drosophila melanogaster*, *V-ATPase B*, *C*, *E*, *G* and *H* are only single gene. Other V-ATPase genes consist of more than two genes, and subunits a and c are both encoded by five genes [7,8]. Mutations of *V-ATPases* lead to the defective development of the egg chambers, eyes and wings in *Drosophila* [9,10,11].

To date, the functions of *V-ATPases* in pests have been studied by RNAi in nine orders (Diptera [12], Lepidoptera [13,14,15], Hemiptera [16,17,18,19,20], Homoptera [21,22], Blattodea [23], Hymenoptera [24], Coleopterans [25,26,27,28,29,30,31,32,33,34,35,36], Thysanoptera [37] and Orthoptera [38,39,40]). Studies in several pests have demonstrated that knockdown of individual *V-ATPase* could be lethal, which makes *V-ATPase* a potential target for RNAi-based pest control. One breakthrough was the development of transgenic maize engineered to express the dsRNA of *V-ATPase A*; these plants showed significant reduction in feeding in the western corn rootworm larva (*Diabrotica virgifera virgifera*) [25]. In addition to lethality, developmental abnormalities have been reported after *V-ATPases* RNAi, such as molting defects in *Periplaneta fuliginosa* for *V-ATPase B* [23]; pupation failure in *Helicoverpa armigera* for *V-ATPase A* [13] and *Aethina tumida* for *V-ATPase A* [31]; and low fecundity in *Peregrinus maidis* for *V-ATPase B* and *D* [21], *Planococcus citri* for *V-ATPase* [22], *Cimex lectularius* for *V-ATPase A* and *E* [16] and *Frankliniella occidentalis* for *V-ATPase B* [37]. In *H*. *armigera*, it was reported that V-ATPase E mediated Cry2Ab binding and toxicity [41]. In *Plutella xylostella*, silencing of *PxVHA-G1* increased the susceptibility of *P. xylostella* to Cry1Ac toxin [42]. All these results indicate that V-ATPases are crucial in the growth and development of insects.

In *Locusta migratoria*, Shi et al. and Liu et al. identified and analyzed the function of two genes encoding V-ATPase subunits c and a in 2022 [39,40]. *LmV-ATPase c* is essential for normal molting and is involved in a systemic RNA interference response [40], while *LmV-ATPase a* is required for the midgut development of locusts [39]. In addition, knockdown of *LmV-ATPase H* caused molting defects in fifth instar nymphs according to Li et al. in 2012 [38], but the authors did not further show the possible reasons for this phenomenon. In this paper, we further identified other V-ATPase subunits and analyzed the expression patterns of LmV-ATPase genes in various tissues and different developmental stages. Injection of dsRNA against each LmV-ATPase gene led to 100% mortality, with abnormalities in the wing and cuticle. The results demonstrated that *V-ATPase* could potentially be a good target for RNAi-based pest management.

## 2. Materials and Methods

### 2.1. Insects

*L*. *migratoria* eggs were supplied by a locust breeding center (Hebei, China) and hatched under the conditions of 30 ± 2 °C, 40 ± 10% RH and an L14 h/D10 h photoperiod in our laboratory. After hatching, the nymphs were fed with fresh wheat seedlings.

### 2.2. Identification and Bioinformatics Analysis of LmV-ATPase Genes

The *D. melanogaster* protein sequences of V-ATPase genes were downloaded from FlyBase (http://flybase.org/), accessed on 8 October 2019. These *V-ATPase* sequences were then used to BLAST the locust transcriptome database (GEZB00000000) [43]. The amino acid sequences were deduced using the ExPASy-translational tool (https://web.expasy.org/translate/), accessed on 8 October 2019. The individual protein sequences of V-ATPases were used for BLASTP to search against *D. melanogaster* homologs to assess their homology degree. The transmembrane domains of V-ATPase proteins were predicted by TMHMM Server v. 2.0 (http://www.cbs.dtu.dk/services/TMHMM/), accessed on 8 October 2019. The molecular weight (Mw) and isoelectric point (pI) were predicted by using the relevant software tools in ExPASy (https://web.expasy.org/compute_pi/), accessed on 8 October 2019.

### 2.3. Total RNA Isolation and First-Strand cDNA Synthesis

To study tissue-specific expression patterns of *LmV-ATPases*, eleven tissues, including samples from the head (HD), integument (IN), foregut (FG), gastric caecum (GC), midgut (MG), hindgut (HG), fat body (FB), hemolymph (HE), trachea (TR), testis (TE) and ovary (OV), were dissected from day 4 of 3rd instar nymphs (N3D4). For analysis of stage-dependent expression patterns of *LmV-ATPases*, whole bodies of nymphs from day 1 of 3rd instar to day 7 of 5th instar (N3D1-N5D7) were collected. All samples were collected as six biological replicates, each with five nymphs. RNAiso^TM^ Plus (TaKaRa, Tokyo, Japan) was used to extract total RNA. The quality and quantity of total RNA were determined on 1.5% (*w*/*v*) agarose gel and a NanoDrop 2000 instrument (Thermo Fisher Scientific, Waltham, MA, USA). First-strand cDNAs were synthesized using 1.5 μg RNA with M-MLV reverse transcriptase (TaKaRa, Tokyo, Japan). Each cDNA was diluted 10-fold for quantitative reverse transcribed (RT)-PCR (RT-qPCR) analysis.

### 2.4. RT-qPCR

The RT-qPCR reactions were performed with SYBR Green qPCR Master Mix (TaKaRa, Tokyo, Japan) according to the manufacturer’s recommendations on a Bio-Rad system (Bio-Rad Laboratories, Hercules, CA, USA). The primer sequences are listed in Table S1. Each 20 μL sample RT-qPCR reaction contained 10 μL of 2× SYBR qPCR Master Mix, 1.6 µL of sense and antisense primer mixture (10 μM), 3 μL of cDNA templates and 5.4 μL of ddH_2_O. The RT-qPCR cycling conditions were as follows: 95 °C for 30 s, followed by 40 cycles of 95 °C for 15 s and 60 °C for 31 s. The relative mRNA levels of *LmV-ATPase* were normalized to the expression of the internal marker gene *β-actin*, which was previously demonstrated to be stably expressed in different tissues and at different stages [44]. A melting curve was verified for each RT-qPCR to determine the specificity of primers. The relative gene expression levels among *LmV-ATPases* were calculated using 2^−ΔCT^ methods. Tukey’s HSD multiple comparison test was used to show the significant differences of the relative expressions *LmV-ATPases* in different tissues and developmental stages in *L. migratoria*.

### 2.5. RNA Interference (RNAi)

To further investigate the functions of each *LmV-ATPase* in *L. migratoria*, we performed RNAi experiments. The forward and reverse primers for dsRNA synthesis were designed using the E-RNAi web service (https://e-rnai.dkfz.de/signaling/e-rnai3/), accessed on 1 December 2019. The T7 promoter (gcgtaatacgactcactatagg) was added at the 5′-end to amplify the dsRNA fragment (Table S2). The cDNA of whole bodies of N3D1 nymphs were used as templates to generate the T7-containing PCR products. Each 50 μL PCR reaction was prepared containing 20 μL of 2× Taq PCR Master Mix (TIANGEN, Beijing, China), 2.0 µL of sense and antisense primers mixture (10 μM), 2 μL of cDNA templates and 21 μL of ddH_2_O. The PCR cycling conditions were as follows: 95 °C for 3 min, followed by 35 cycles of 94 °C for 15 s, 60 °C for 30 s and 72 °C for 50 s. The PCR products were sub-cloned and sequenced via Sanger sequencing. The double-stranded RNA (dsRNA) targeting each *LmV-ATPase* and green fluorescent protein (GFP) gene (negative control) were synthesized in vitro by using T7 RiboMAX™ Express RNAi System (Promega, Madison, WI, USA). The dsRNAs were dissolved in nuclease-free water, and the integrity and specificity were analyzed on a 2% agarose gel. The concentrations of the dsRNA were determined and adjusted to 2 μg/μL.

Using a 25 μL microinjector (Gaoge Co., Ltd., Shanghai, China), aliquots of 6 µg dsRNA of each *LmV-ATPase* were injected into the hemocoel between the second and third abdominal segments of N3D1 nymphs. The controls were injected with equal amounts of ds*GFP*. For each treatment of the RNAi experiment (ds*GFP*, ds*LmV-ATPase A*, ds*LmV-ATPase B*, ds*LmV-ATPase C*, ds*LmV-ATPase D*, ds*LmV-ATPase E*, ds*LmV-ATPase F*, ds*LmV-ATPase G*, ds*LmV-ATPase c*″, ds*LmV-ATPase d* and ds*LmV-ATPase e*), more than 50 locusts were used. Twenty-four hours after dsRNA injection, whole bodies were used to extract total RNA. Four insects were used in each biological replicate, and four biological replicates were set. The relative expression levels of each *LmV-ATPase* were detected by RT-qRCR, as described above. Student’s *t*-test was used for the gene silencing analysis. The phenotypes of the remaining dsRNA-injected nymphs were observed

### 2.6. Hematoxylin–Eosin (H&E) Staining

To further investigate the roles of *LmV-ATPase* knockdown on the cuticle and wing development, paraffin sections were prepared and stained with H&E. The cuticle were collected from N3D5 nymphs after treatment with ds*LmV-ATPase c*″ and ds*LmV-ATPase e* or ds*GFP*. The wings were collected from N4D1 nymphs after injection of ds*LmV-ATPase A* and ds*LmV-ATPase B* or ds*GFP*. The samples were prepared for H&E staining as described previously [45]. Briefly, cuticles and wings were fixed in 2.5% glutaraldehyde and then washed, dehydrated and made transparent; later, they were treated with wax, embedded in paraffin and stained with H&E (Servicebio, Wuhan, China). Images were obtained using an Olympus BX51 (Olympus, Tokyo, Japan).

### 2.7. Transmission Electron Microscopy (TEM)

To further investigate the roles of *LmV-ATPase* knockdown at the ultrastructural level, TEM was performed with nymphs treated with ds*LmV-ATPase c*″ and ds*LmV-ATPase e* or ds*GFP*, as described previously [45]. The abdominal cuticles and wings of N3D5 or N4D1 nymphs were dissected, respectively. Briefly, these tissues were processed into 80 nm ultrathin sections, following which sections were collected on copper grids and stained with uranyl acetate and lead citrate. The images were obtained using a JEM-1200EX TEM (JEOL, Tokyo, Japan).

## 3. Results

### 3.1. Identification and Characterization of the LmV-ATPase Genes

**Figure 1:**
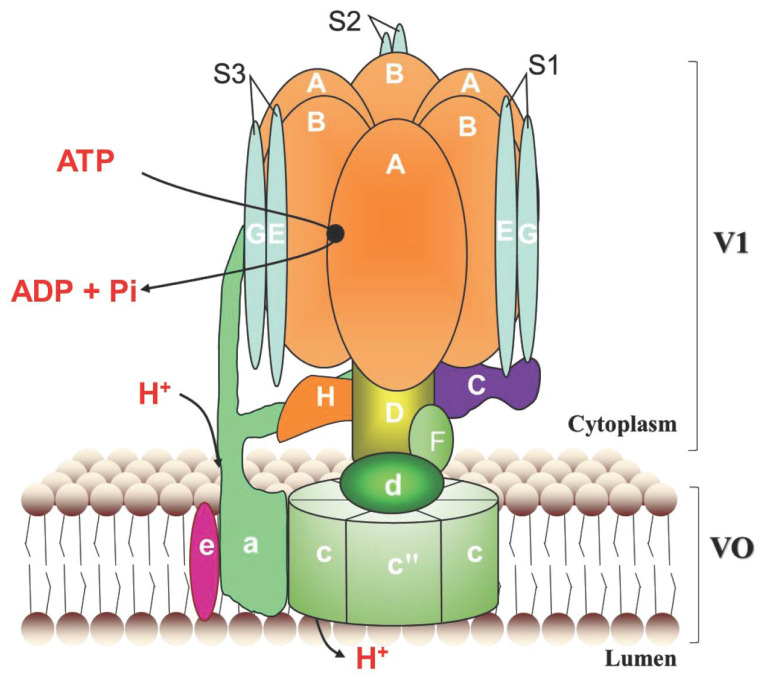
The structure diagram of V-ATPase in insects. A holoenzyme V-ATPase consists of two functional complexes, the cytoplasmic V1 and the membrane-embedded Vo. V1 is composed of subunits A–H and is responsible for ATP hydrolysis. The Vo domain, which carries out protons transport, is composed of subunits a, d, e, c, and c″.

A holoenzyme V-ATPase consists of V1 and Vo domains. V1 is composed of subunits A-H, Vo is composed of subunits a, d, e, c, and c″, shown in Figure 1. We obtained ten V-ATPase genes, which were named as *LmV-ATPase A*, *B*, *C*, *D*, *E*, *F*, *G*, *c*″, *d* and *e*, respectively. These genes contain 12, 10, 9, 6, 4, 1, 4, 7, 7 and 4 exons according to the genomic database [46], respectively (Figure S1). These genes encode products of 615, 500, 423, 385, 250, 123, 118, 208, 348 and 84 amino acid residues, respectively. The deduced Mw and pI of LmV-ATPases varied much from 9.2 to 96.0 kDa, and 4.93 to 9.66. Among them, LmV-ATPase c″ and LmV-ATPase e have 5 and 2 transmembrane regions, respectively (Table 1).

### 3.2. Expression Patterns of LmV-ATPase Genes

**Figure 2:**
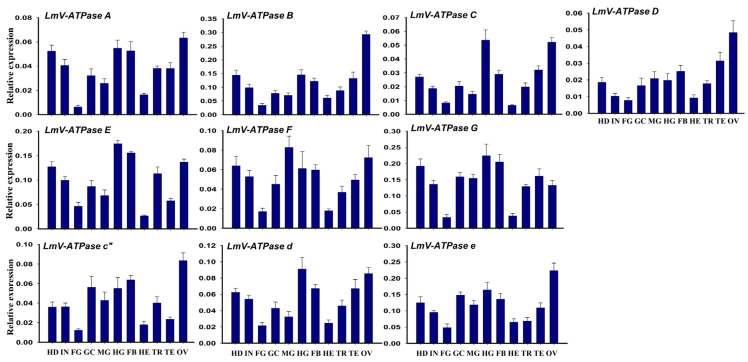
Tissue-dependent expression patterns of *LmV-ATPases*, examined using RT-qPCR. HD: head; IN: integument; FG: foregut; GC: gastric caecum; MG: midgut; HG: hindgut; FB: fat body; HE: hemolymph; TR: trachea; TE: Testis; OV: Ovary. All data are shown as means ± SD. Error bars represent SD for six biological replicates. (One-way ANOVA with Tukey’s test, *p* < 0.05).

The tissue-dependent expression patterns of *LmV-ATPases* from 3rd instar nymphs were explored via RT-qPCR. As shown in Figure 2, the transcripts of *LmV-ATPases* were highly expressed in the head (HD), integument (IN), gastric caecum (GC), midgut (MG), hindgut (HG), fat body (FB), trachea (TR) and ovary (OV), but showed low expression levels in foregut (FG) and hemolymph (HE).

**Figure 3:**
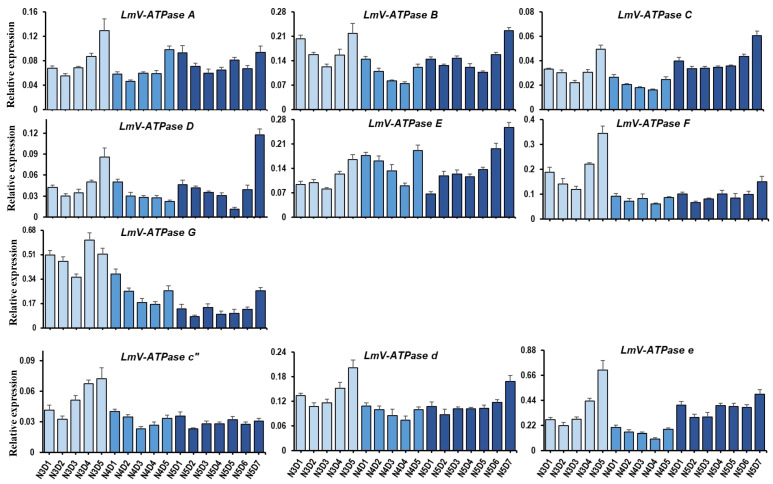
The expression of *LmV-ATPases* from 3rd to 5th instar nymphs, examined using RT-qPCR. All data are shown as means ± SD. Error bars represent SD for six biological replicates (one-way ANOVA with Tukey’s test, *p* < 0.05). Different colors indicate different nymphal stages.

The developmental expression patterns of *LmV-ATPases* in the whole bodies from 3rd instar to 5th instar nymphs were explored using RT-qPCR. These *LmV-ATPases* were expressed in the examined stages, which were from 3rd to 5th instar nymphs. In general, the expression of *LmV-ATPases* showed a relatively low level in the early and middle days of each stage, and then increased greatly before molting (Figure 3).

### 3.3. Effect on Locust Survival After LmV-ATPase Genes Knockdown

**Figure 4:**
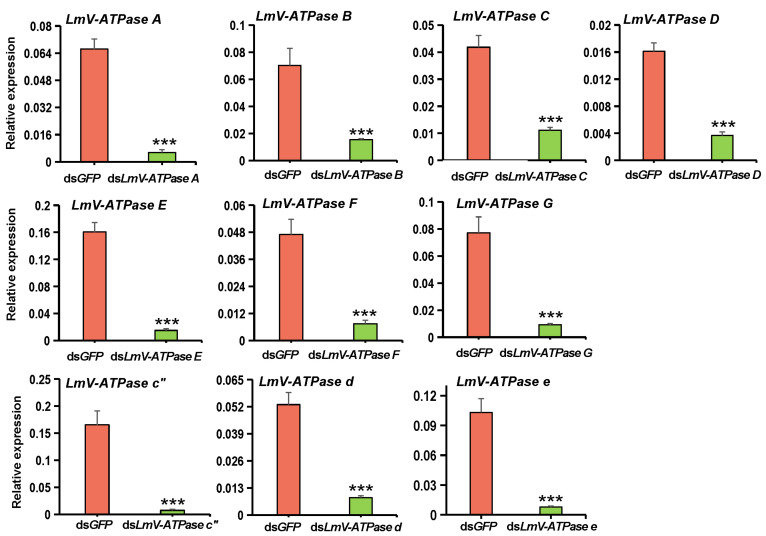
The expression of each *LmV-ATPase* after RNAi, examined using RT-qPCR. (Student’s *t*-test; ***, *p* < 0.001).

To further explore the effects of *LmV-ATPase* genes knockdown on the growth and development of locusts, RNAi was performed against each *LmV-ATPase* gene in newly molted 3rd instar nymphs. Compared with the controls, the transcript levels of *LmV-ATPase A*, *B*, *C*, *D*, *E*, *F*, *G*, *c*″, *d* and *e* decreased significantly after 24 h post-dsRNA injection (Figure 4, Table 2).

**Figure 5:**
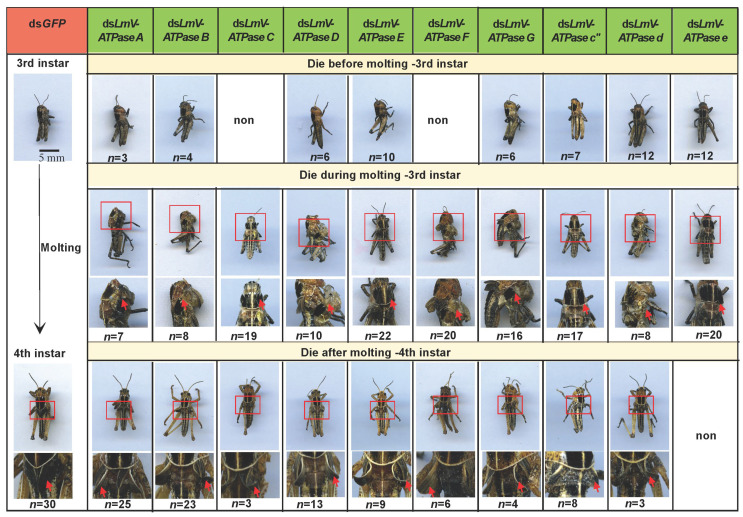
Phenotypes after injection dsRNA against each *LmV-ATPase* (arrows). Insects injected with ds*LmV-ATPase* died before molting, during molting and after molting.

As shown in Table 2, compared with the control, the accumulative mortality of insects injected with ds*LmV-ATPase A*, ds*LmV-ATPase B*, ds*LmV-ATPase C*, ds*LmV-ATPase D*, ds*LmV-ATPase E*, ds*LmV-ATPase F*, ds*LmV-ATPase G*, ds*LmV-ATPase c*″, ds*LmV-ATPase d* and ds*LmV-ATPase e* was 92.0%, 89.7%, 76.7%, 93.5%, 95.3%, 83.9%, 72.2%, 97.0%, 82.7% and 91.4%, respectively. Amongst the dead locusts in the RNAi group, some locusts died before molting to 4th instar nymphs and some locusts died during the molting stage; specifically, these locusts just had an opening at the back of the old cuticle. Some locusts had a near-complete molting, but they could not shed the old cuticle completely and finally died. Furthermore, some locusts died after molting to 4th instar nymphs with 1–2 days, but their wings had severe defects. In particular, the wings were curled (Figure 5).

### 3.4. Effect on the Locust Cuticle After Knockdown LmV-ATPase c″ and LmV-ATPase e

**Figure 6:**
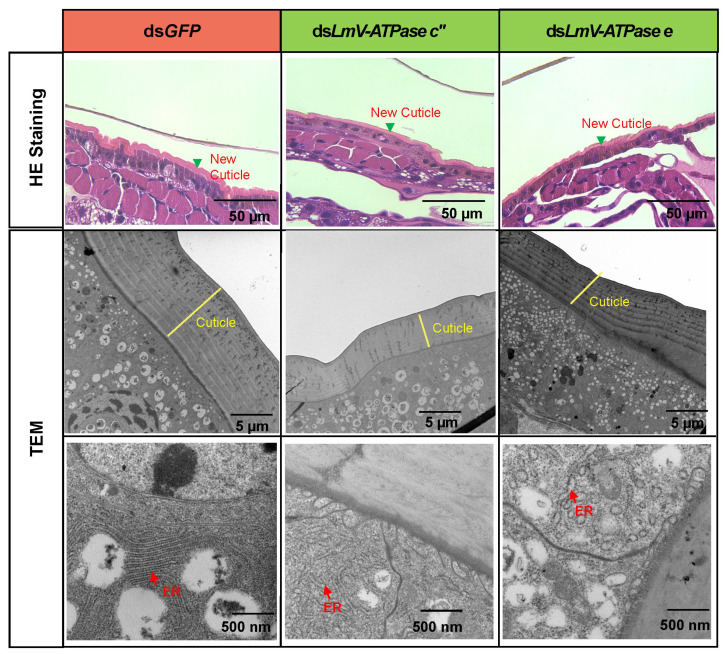
Histology and TEM analysis of the integument after *LmV-ATPase c*″ and *LmV-ATPase e* RNAis. The integument of N3D5 nymphs were used for histology and TEM analysis after injection of ds*GFP* or ds*LmV-ATPase c*″ and ds*LmV-ATPase e*. ER: endoplasmic reticulum.

To observe the effects of ds*LmV-ATPase c*″ and ds*LmV-ATPase e* on the cuticle development of locusts, we observed the changes in cuticles in the paraffin sections. The integument of ds*LmV-ATPase c*″*-*, ds*LmV-ATPase e-* and ds*GFP*-injected 3rd instar nymphs were prepared at day 5 and stained with H&E. In the ds*GFP*-injected controls, a new cuticle was normally formed, and the old cuticle was detached from the epidermal cells and shed and replaced by the new cuticle during the molting process (Figure 6). Compared with those of the control, significantly fewer new cuticles were formed in the ds*LmV-ATPase c*″ and ds*LmV-ATPase e* treatments (Figure 6).

To observe the effects of ds*LmV-ATPase c*″ and ds*LmV-ATPase e* on the cuticle of locusts at the ultrastructural level, TEM was further performed. As shown in Figure 6, in the ds*GFP*-treated nymphs, the new cuticle was normally synthesized with multiple laminaes visible, but the new cuticle was obviously thinner in the ds*LmV-ATPase c*″ and ds*LmV-ATPase e*-injected locusts. In addition, the endoplasmic reticulum showed a normal form in the controls. However, in the ds*LmV-ATPase c*″-treated and ds*LmV-ATPase e*-treated nymphs, endoplasmic reticulum swelling was evident.

### 3.5. Effect on the Locust Wing After Knockdown LmV-ATPase A and LmV-ATPase B

**Figure 7:**
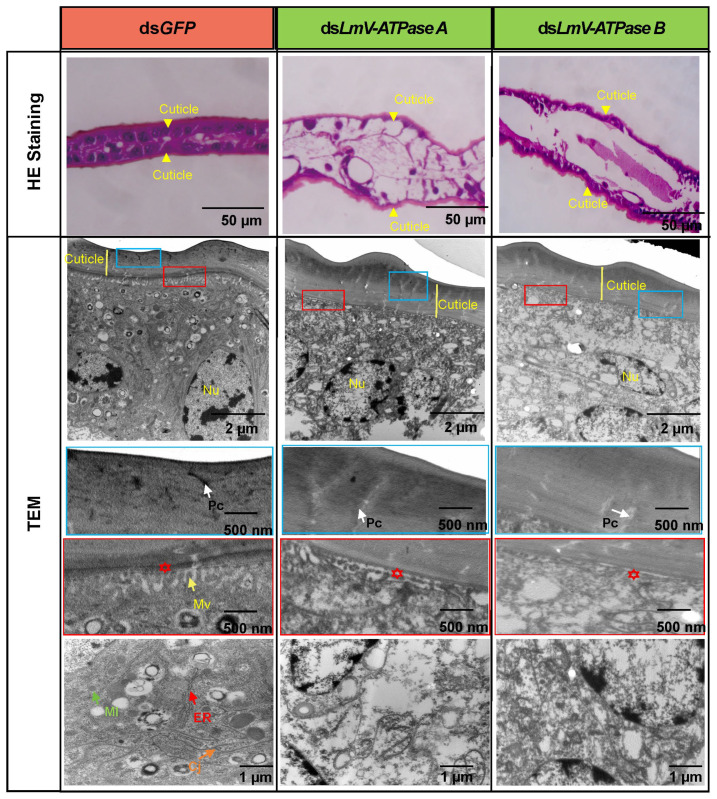
Histology and TEM analysis of the wings after *LmV-ATPase A* and *LmV-ATPase B* RNAi. The wings were dissected from N4D1 nymphs after injection of ds*GFP* or ds*LmV-ATPase A* and ds*LmV-ATPase B*. Yellow arrow, orange arrow, white arrow and green arrow indicate microvilli (MV), cell junction (CJ), pore canal (Pc) and mitochondria (MI), respectively. Red asterisks indicate the space that loss of *LmV-ATPase A* and *LmV-ATPase B* leads to detachment of the cuticle from epithelial cells.

As shown in Figure 5, compared with the control, approximately 65.8% and 58.9% of the wings were defective after knockdown of *LmV-ATPase A* and *LmV-ATPase B*, respectively. In order to study the effects on the wing formation after *LmV-ATPase* RNAi, we observed the structure of the wing of nymphs injected with ds*GFP*, ds*LmV-ATPase A* and *LmV-ATPase B* ( Figure 7). The H&E staining results showed that the ventral cuticle and dorsal cuticle of the wings cannot adhere to each other in the ds*LmV-ATPase A-* and *LmV-ATPase B*-injected insects. Although the thickness of the wing cuticle was normal in both ds*LmV-ATPase A* and *LmV-ATPase B*-treated insects, further TEM analysis revealed that the pore canals showed light electron density, and the wing epithelial cells contained cell debris. In particular, the short microvilli of apical plasma membranes, mitochondria, cell junctions and endoplasmic reticulum of the wing epithelial cells had different degrees of degradation.

## 4. Discussion

Published reports indicate that V-ATPases play key roles in many biological processes. However, the molecular characterization and functions of V-ATPase are largely unknown in the insect pest *L. migratoria*. In the present study, we demonstrated that *LmV*-ATPase genes are essential for the survival of locusts.

### 4.1. Identification and Molecular Characterization of the Locust V-ATPase Genes

Previous studies have reported the presence of *LmV-ATPase H*, *a* and *c* in *L. migratoria* [38,39,40]. In this study, we obtained ten other V-ATPase genes (*LmV-ATPase A*, *B*, *C*, *D*, *E*, *F*, *G*, *c*″, *d* and *e*). Our data revealed that *LmV-ATPases* were highly expressed in many tissues, including the head, integument, gastric caecum, midgut, hindgut, fat body, trachea and ovary (Figure 3). Furthermore, we found that *LmV-ATPases* were widely expressed in the examined third to fifth instar nymphs (Figure 3). In agreement with our results, in *D. melanogaster*, in situ hybridization shows that *Vha44*, *Vha26* and *VhaSFD* (encoding C, E and H subunits, respectively) are expressed in multiple tissues (the Malpighian tubules, midgut, hindgut, ovary, testes and rectum) and are expressed in the embryos, larvae, pupae and adults [8]. In *Hyphantria cunea*, *HcV-ATPase A* and *C* were found to be expressed in the head, foregut, midgut, hindgut, Malpighian tubules, epidermis and fat body and developmental stages examined (from the first instar to pupae) [15]. In *Henosepilachna vigintioctopunctata*, *HcV-ATPase A*, *B*, *C*, *E*, *F*, *H*, *a* and *d* are widely expressed in the hindgut and Malpighian tubules and found in the eggs, first to fourth instar larvae, prepupae, pupae and adults [32,33,34,35,36]. The expression data provide experimental evidence to support that V-ATPase may have diverse functions and play important roles throughout all developmental stages.

### 4.2. LmV-ATPases Are Essential for the Survival of Locusts

Considering the key roles of *V-ATPase*, targeting V-ATPase genes for purposes of pest control has been reported in several insects. For example, the knockdown of *V-ATPase A* was lethal in *Diabrotica virgifera virgifera* (Coleoptera: Chrysomelidae) [26,27], *Bemisia tabaci* (Hemiptera: Aleyrodidae) [17], *Aethina tumida* (Coleoptera: Nitidulidae) [31] and *Hyphantria cunea* (Lepidoptera: Arctiidae) [15], as was subunit B in *Frankliniella occientalis* (Thysanoptera: Thripidae) [37], *Peregrinus maidis* (Hemiptera: Delphacidae) [21] and *Liriomyza trifolii* (Diptera: Agromyzidae) [12]; subunit C in *H. cunea* [15]; subunit D in *L. trifolii* [12] and *P. maidis* [21]; subunit E in *Solenopsis invicta* (Hymenoptera:Formicidae) [24]; subunit F in *Henosepilachna vigintioctopunctata* (Coleoptera: Coccinellidae) [33]; and subunit H in *H. vigintioctopunctata* [35] and *Aphis gossypii* (Homoptera:Aphididae) [19]. Most functional studies on *V-ATPases* of insects have focused on genes encoding subunits of the V1 complex, and only a few studies have addressed genes encoding subunits of the Vo complex. In *H. vigintioctopunctata*, the knockdown of *HcV-ATPase a* and *d* significantly increased mortality [47].

We further explored the functions of *LmV-ATPase* by injecting dsRNA into N3D1 locusts. Our results also demonstrated that these LmV-ATPase genes are required for the survival of locusts. As shown in Table 2, the mortality of insects injected with dsRNA against each *LmV-ATPase* varied from 72.2% to 97.0%. These studies imply an important function of *V-ATPase* among insects, but the mortalities were different among species. Undoubtedly, these data suggest that V*-ATPases* may be suitable targets in pest control.

### 4.3. LmV-ATPases Are Involved in the Cuticle and Wing Development

Targeting of *LmV-ATPases* by RNAi led to developmental defects, notably the *LmV-ATPases*-injected nymphs did not moult, and therefore died within the old cuticle. This is in line with the previous study which reported molting defects in *L. migratoria* after knockdown *V-ATPase H* and *c* [39,40]. Similarly, RNAi of the V-ATPase-B gene in *P. fuliginosa* caused molting defects, with wrinkled cuticles of thoraxes and abdominal segments, and growth inhibition [23]. We further demonstrated that the failure of molting was partially the result of an inability of nymphs to synthesize normal new cuticles after *LmV-ATPase c*″ and *LmV-ATPase e* RNAis (Figure 6). We then observed the ER of the integument by TEM analysis, showing that it loses its tubular shape and becomes spherical in *L. migratoria*. The change in ER is also found after blocking coat protein II (COPII) genes involved in vesicle transport in *L. migratoria* and *Drosophila* [48,49]. COPII is a coated vesicle which can transport newly synthesized proteins from the ER to the Golgi [50]. V-ATPase is also essential for vesicular trafficking in *Caenorhabditis elegans* [51]. It is possible that V-ATPases might regulate vesicular trafficking as reported in *C. elegans*.

We then found that the wings were defective after *LmV-ATPase A* and *LmV-ATPase B* knockdown, respectively. The ventral cuticle and dorsal cuticle of the wings cannot adhere to each other, and mitochondria, endoplasmic reticulum and cell junctions had different degrees of degradation (Figure 7). Our findings suggest a novel function of *LmV-ATPase A* and *LmV-ATPase B* in wing development. In *Drosophila*, mutants of Vha100-1 to Vha100-5 (different isoforms of V-ATPase subunit a) also resulted in different defects in the development of fly wings [52]. These previous studies further demonstrated that Vha100-2 is essential for wing cuticle formation, while Vha100-4 plays important roles in wingless signaling activation. As V-ATPases are highly conserved multi-subunit complexes, it is possible that LmV-ATPases may function during the wing formation. However, the exact mechanism of V-ATPases in the development of the cuticles and wings requires further investigation.

## 5. Conclusions

**Figure 8:**
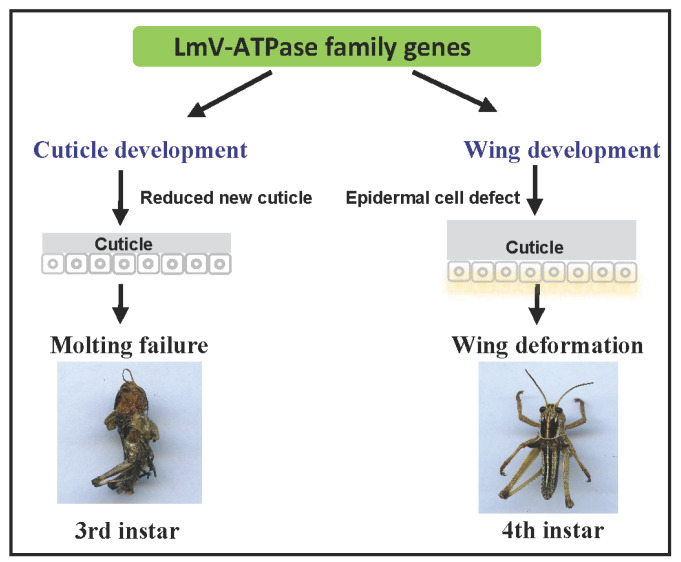
Model of the locust cuticle and wing metamorphosis mediated by *LmV-ATPase. LmV-ATPases* play key roles in the development of the epidermal cuticle and wing cuticle in locusts.

In summary, we first identified LmV-ATPase genes (LmV-ATPase-*A*, *B*, *C*, *D*, *E*, *F*, *G*, *c*″, *d* and *e*), and among them, LmV-ATPase c″ and LmV-ATPase e were found to contain transmembrane regions. Then, RT-qPCR results further showed that LmV-ATPase genes might exert functions in multiple tissues and across developmental stages. The knockdown of each LmV-ATPase gene using RNAi resulted in defective development of the epidermal cuticle and the epidermis of the wings (Figure 8). These results showed the possibility of leveraging *LmV-ATPase* in *L. migratoria* control.

## Figures and Tables

**Table 1:** Analysis of LmV-ATPase proteins. The amino acid residues predicted to function as transmembrane domains (TM) are indicated for each protein.

Name	CDS (bp)	Amino Acids	Mw (KDa)	pI	TM (Position; aa)
LmV-ATPase A	1848	615	68.2	5.11	-
LmV-ATPase B	1503	500	55.7	5.33	-
LmV-ATPase C	1158	385	44.4	8.16	-
LmV-ATPase D	753	250	28.2	9.63	-
LmV-ATPase E	684	227	26.2	8.51	-
LmV-ATPase F	372	123	13.8	5.92	-
LmV-ATPase G	357	118	14.0	9.66	-
LmV-ATPase H	1590	529	60.8	5.54	-
LmV-ATPase c″	627	208	21.7	8.87	5–27; 47–69; 89–111; 137–159; 172–194
LmV-ATPase d	1047	348	39.6	4.93	-
LmV-ATPase e	255	84	9.2	9.35	4–26; 33–51

**Table 2:** Summary of the RNAi analysis of LmV-ATPase genes.

Gene Name	Gene Silencing Efficiency	Nymphs (*n*)	Die Before Molting	Die During Molting	Die After Molting	The Accumulative Mortality
*LmV-ATPase A*	90.2%	38	7.8%	18.4%	65.8%	92.0%
*LmV-ATPase B*	78.1%	39	10.3%	20.5%	58.9%	89.7%
*LmV-ATPase C*	73.3%	32	non	59.3%	9.4%	76.7%
*LmV-ATPase D*	77.2%	31	19.3%	32.3%	41.9%	93.5%
*LmV-ATPase E*	90.6%	43	23.2%	51.2%	20.9%	95.3%
*LmV-ATPase F*	84.2%	31	non	64.5%	19.4%	83.9%
*LmV-ATPase G*	88.2%	36	16.7%	44.4%	11.1%	72.2%
*LmV-ATPase c”*	95.5%	33	21.2%	51.5%	24.3%	97.0%
*LmV-ATPase d*	84.2%	29	44.8%	27.6%	10.3%	82.7%
*LmV-ATPase e*	92.5%	35	34.3%	57.1%	non	91.4%

non: no nymph died.

## Data Availability

The data that support the findings of this study are available on reasonable request from the first and corresponding author.

## Supplementary Materials

The following supporting information can be downloaded at: https://www.mdpi.com/article/10.3390/genes16020145/s1. Table S1: Primer information for RT-qPCR; Table S2: Primer information for dsRNA synthesis; Figure S1: Schematic diagram of gene structures of LmV-ATPase genes.
