# Supplementary material for: Vacuolar (H+)-ATPase Genes Are Essential for Cuticle and Wing Development in Locusta migratoria
Source: Genes (Basel). 2025 Jan 24;16(2):145. doi: 10.3390/genes16020145 (PMC11854941; doi:10.3390/genes16020145)
Supplement: Supplementary file 1 [file genes-16-00145-s001.zip › genes-3389630-supplementary.pdf]

genes

*Article*

# The vacuolar (H+ )-ATPase genes are essential for the cuticle and wing development in *Locusta migratoria*

**Xiaojian Liu [1]*, Xiaoyu Liang [1], Xuekai Shi [2] and Jianzhen Zhang [1]***

[1] Shanxi Key Laboratory of Nucleic Acid Biopesticides; Research Institute of Applied Biology, Shanxi University, Taiyuan, Shanxi 030006, China

[2] College of Biological Sciences and Technology, Taiyuan Normal University, Jinzhong, Shanxi 030619, China

* Correspondence: Xiaojian Liu, Email addresses: xiaojianliu@sxu.edu.cn; Jianzhen Zhang, Email addresses: zjz@sxu.edu.cn

**Table S1.** Primer information for RT-qPCR.

| Name | Sequence (5′-3′) | Length (bp) |
|---|---|---|
| *β-actin* | F: CGAAGCACAGTCAAAGAGAGGTA | 156 |
| | R: GCTTCAGTCAAGAGAACAGGATG | |
| *LmV-ATPase A* | F: CAAGGATGGAGAAGCGAAGAT | 189 |
| | R: GGCAAAGGAATGCTCAGGTGT | |
| *LmV-ATPase B* | F: TGACTCAAGACACCCTCAAACTA | 99 |
| | R: AGTCCCTTTCTAACACAACCACC | |
| *LmV-ATPase C* | F: ATGCTGCTTCACCCACACAAAAA | 137 |
| | R: CCAAACCCAAGTCCTGGTATCTC | |
| *LmV-ATPase D* | F: CATAGCCAGAGCCAAAGCC | 134 |
| | R: CACCTAATGCCTAGAACAA | |
| *LmV-ATPase E* | F: ATTGTTGATAGCATCTTGCCAGC | 179 |
| | R: TCCAGCCGTGCTTCCAAAGTATT | |
| *LmV-ATPase F* | F: AAAGATTCGATATTACGTAGGGCA | 180 |
| | R: ACACCACTTTCATTTGAGCCTTAG | |
| *LmV-ATPase G* | F: CAAGCCCGAACTCCATAAGAAC | 208 |
| | R: TCTGTATGATTATCCCACCACC | |
| *LmV-ATPase c″* | F: AACTCAGCGTTATTTGTCA | 117 |
| | R: TTACTGCTTGTCTCCCATT | |
| *LmV-ATPase d* | F: TACAGTGCTTTATTTGAAGGTGC | 167 |
| | R: ATGTTACGGCATTCTTGTTCCTT | |
| *LmV-ATPase e* | F: GGCTCAAATGAATCCTTTG | 184 |
| | R: GACAGTTCCCTTCTGAAATA | |

**Table S2.** Primer information for dsRNA synthesis

| Name | Sequence (5´-3´) | Length (bp) |
|---|---|---|
| *LmV-ATPase A* | F: taatacgactcactatagggGCAAACACATCAAACATGCC | 500 |
| | R: taatacgactcactatagggACCTTTGTACGCAGTGGGAC | |
| *LmV-ATPase B* | F: taatacgactcactatagggTGACAGCTGCTGAATTCCTG | 488 |
| | R: taatacgactcactatagggCTCACCAACAACAGCCTTCA | |
| *LmV-ATPase C* | F: taatacgactcactatagggAAGGCAACCTGCAAAATCTG | 500 |
| | R: taatacgactcactatagggGGGCAGTCCATACCTCAGAA | |
| *LmV-ATPase D* | F: taatacgactcactatagggGCGCAGATGTTAATGAAAGC | 516 |
| | R: taatacgactcactatagggGTAGGCAAGAGTACGTTCA | |
| *LmV-ATPase E* | F: taatacgactcactatagggCTAAGCGATGCTGATGTCCA | 396 |
| | R: taatacgactcactatagggTACAGTGACATTCGGCTCCA | |
| *LmV-ATPase F* | F: taatacgactcactatagggACATTCGGCTTACAAAGGGA | 297 |
| | R: taatacgactcactatagggTAAGGATGGTCCTTTGACGG | |
| *LmV-ATPase G* | F: taatacgactcactatagggAAGGGCAGCAGAGAAGGTTT | 240 |
| | R: taatacgactcactatagggATCAACTTTTCCTTCCGCTG | |
| *LmV-ATPase c″* | F: taatacgactcactatagggCTAGCGACGCTTTCGTCTG | 461 |
| | R: taatacgactcactatagggGCACCAGATCCCACAATTC | |
| *LmV-ATPase d* | F: taatacgactcactatagggCGGGAGCTTTTTAGCAAATG | 457 |
| | R: taatacgactcactatagggTCAGCTGTTGTTCCACCAAG | |
| *LmV-ATPase e* | F: taatacgactcactatagggCCGCAGCTACGCCTATTATT | 225 |
| | R: taatacgactcactatagggCATTCCCTGGACATAATCGC | |
| *GFP* | F: taatacgactcactatagggGACGTAAACGGCCACAAGTT | 496 |
| | R: taatacgactcactatagggTGTTCTGCTGGTAGTGGTCG | |

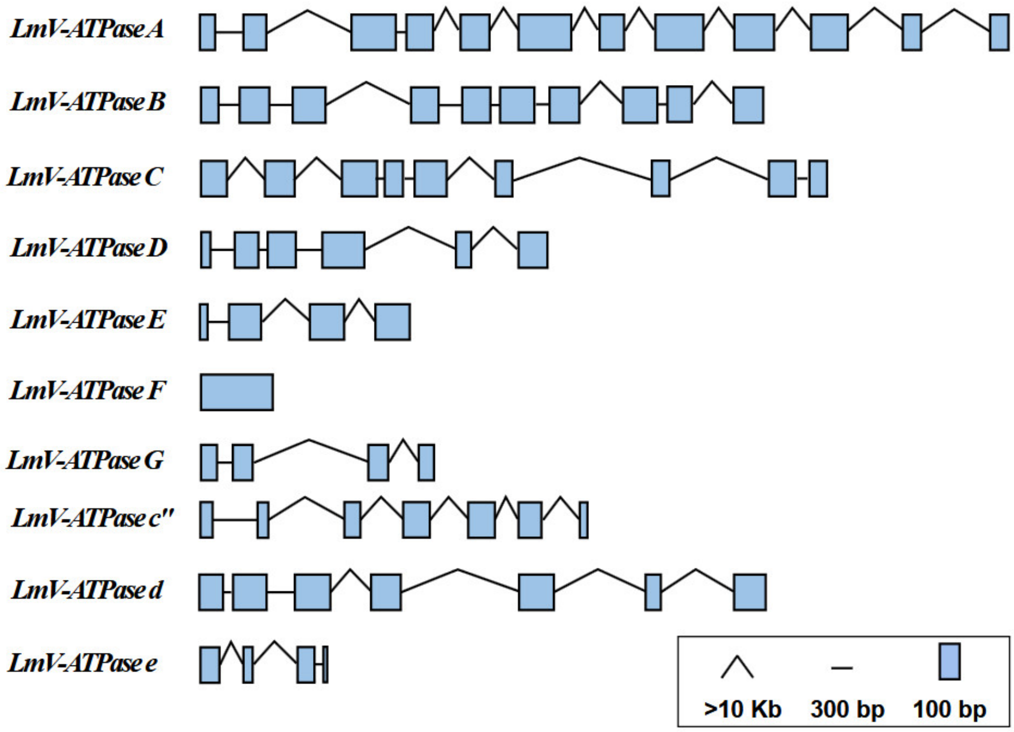


**Figure S1.** Schematic diagram of gene structures of *LmV-ATPase* genes.
